# School failure in students who are normal-hearing or deaf: with or without cochlear implants

**DOI:** 10.1186/s40064-016-1927-9

**Published:** 2016-02-29

**Authors:** Ivone Duarte, Cristina Costa Santos, Guilhermina Rego, Rui Nunes

**Affiliations:** Department of Social Sciences and Health, Faculty of Medicine, University of Porto, Alameda Prof. Hernâni Monteiro, 4200-319 Porto, Portugal; Department of Health Information and Decision Sciences, Centre for Research in Health Technologies and Information Systems (CINTESIS), Faculty of Medicine, University of Porto, Porto, Portugal

**Keywords:** Cochlear implant, Children, Adolescent, Deafness, Hearing loss, Normal hearing, School, School failure

## Abstract

To evaluate the impact of cochlear implants on the school failure of deaf who attend mainstream classes by comparing them to their normal-hearing peers as well as deaf without cochlear implants. This case–control study included participants aged 8–18 years. The number of school years failed was obtained from school records. The greatest differences in achievement levels were found between hearing students and those who were deaf without cochlear implants. Cochlear implants provide educational opportunities for hearing-impaired students, yet those without cochlear implants remain at a great disadvantage. These findings suggest that measures promoting greater equity and quality for all deaf students allow achievement levels closer to those of the not impaired.

## Background

The education of deaf children is a complex problem that manifests itself at different levels. There is not always a clear distinction among the methodological aspects, purposes of action and philosophical, sociological, and political options. Today, the right to public education for all students is not justified simply because it is effective but because it distributes the costs of special schools, reflects the desires of parents, and most of all defends the child´s dignity as a free human being with equal rights.

Thus, education should contribute to the full development of the human being, and each person should become capable of independent and critical thinking, forging his or her own judgment as he or she considers the available options in life. Today, more than ever before, education provides humans with the freedom of thought, judgment, emotions, and imagination required to develop talent and remain, as much as possible, autonomous and participative citizens.

In this sense, and as each society in the various regions of the world face political, economic, social, and cultural challenges, there is an increase in international concern regarding the objectives and content of education. The implementation of extended educational opportunities in effective development for the individual or society depends ultimately on people actually learning, that is, acquiring useful knowledge, reasoning skills and values. Consequently, basic education should focus on the acquisition of actual learning outcomes rather than exclusively on enrolment, established programs, and fulfilment of graduation requirements. For deaf people, as for other citizens, education is critical for employment and social participation in general.

When we consider education, we inevitably also think of educational success, which can be measured in many ways. As is true in many other countries, the success of the Portuguese education system is measured by the outcome of student assessments. The results obtained by this appraisal system can be affected by several factors that interfere, either directly or indirectly, with the final outcome.

Portuguese students are subjected to two evaluation processes, internal summative assessment and external summative assessment. Internal summative assessment occurs in the 1st, 2nd and 3rd grades, and the teachers and school management bodies are responsible for the assessment. External summative assessment occurs in the 4th, 6th and 9th grades and is intended to assess the student’s level of achievement through the use of national evaluation criteria. Summative assessment determines whether a student progresses or is retained. Students with permanent special educational needs can have curricular adaptations on their educational background, and although they take the same external summative assessment tests as the other students, current legislation provides for special assessment allowances, such as extra time for the exam and alternative means of communication, that may benefit such children. Furthermore, children and young people with permanent special educational needs may attend the school with the most appropriate resources (i.e., reference school) regardless of their area of residence and can choose the subjects in which they enroll from 4th grade and on. Deaf children also have the right to bilingual education (Decree-Law No. 3/2008; Legislative Order No. 24-A/2012).

Failure in school can have many lifelong consequences. Grade retention reduces self-esteem and alters peer group formation. It has a negative impact on measures of social adjustment, behavior, self-competence, and attitudes toward school and can cause considerable stress for students. When a grade must be repeated, students perceive it as failure, and some students who fail a grade are more likely to engage in health-impairing behaviors, such as alcohol and drug abuse. Failing students move from classes with their peers to ones with younger students.

The causes of school failure are numerous and usually not the result of a single factor. Social, psychological, behavioral, and academic difficulties and school and health conditions are among the factors that impair academic performance. One in five children who repeat a grade in school has a disability (Byrd 2005; Kamal and Bener 2009). Failure in school is also related to the degree of parental involvement, which plays a vital role in academic performance, as well as the drop-out rate and the amount of money spent on resources (i.e., a failing student costs extra money).

As reported in several studies, children with profound and severe deafness benefit considerably from cochlear implants (Peixoto et al. 2013), and most of these children integrate into mainstream schools (Archbold et al. 2002; Clark 2003). Although the cochlear implant does not transform a deaf child into a normal-hearing child, it helps deaf students make gains despite their remaining educational needs and challenges (Chute and Nevins 2006; Nevins and Chute 1995).

Venail et al. (2010) concluded that children with cochlear implants were more likely to fail early grades in school but ultimately achieve educational and employment levels similar to their normal-hearing peers. They submit that in order to minimize these delays and improve academic success in mainstream education, early oral education and cochlear implantation are important. Other studies have indicated that the educational level of young people with cochlear implants does not differ from that of the normal-hearing population (Huber et al. 2008). Another study involving 41 participants with cochlear implants found that these individuals reached high levels of educational achievement and reported very high levels of satisfaction with life, comparable to those of adults with normal hearing (Spencer et al. 2012).

One of the final objectives of a pediatric cochlear implant program is to provide access for those with severe and profound deafness to an education similar to that of their normal-hearing peers through mainstream education. Many studies report that while there is a trend toward mainstream education for students with cochlear implants, the majority of these students are rated poorly in the area of communication by their teachers and perform below average overall (Nevins and Chute 1995; Mukari et al. 2007).

In this paper, we compared children and adolescents with cochlear implants with their normal-hearing peers as well as deaf students without cochlear implants with respect to the percentage of repeated school years.

## Methods

### Study design

This case–control study included 24 deaf children and adolescents with cochlear implants, 24 deaf children and adolescents without cochlear implants, and 24 normal-hearing children and adolescents aged 8–18 years who attended school in Portugal. The students were matched by gender and school year.

### Setting

The setting was Northern Portugal, where deaf students were attending the same schools as normal-hearing students. The Ministry of Education authorized the study under Order no. 15847/2007. The data were collected during the 2010–2011 school year.

The data characterizing the sample, such as etiology and age at deafness diagnosis, were collected through semi-structured interviews with the parents. The data regarding the number of failures (repeated school years) were obtained from school records.

### Participants

Of the 72 children and adolescents invited to participate, 61 (84.7 %) consented. Twenty of the children/adolescents with profound hearing loss had an cochlear implant (unilateral), and twenty-four of the children/adolescents with profound/severe hearing loss had conventional hearing aids and/or no implants. Both groups had sensorineural bilateral hearing loss. Seventeen individuals with normal hearing also participated in the study. Among those with implants, the age of implantation ranged from 2 to 5 years. All of the children who received implants before beginning school had used them for 3 or more years. All of the deaf children had hearing parents. All of the participants had normal intellectual development, were between the ages of 8 and 18 years, and attended school in Portugal. The participants were matched by gender and school year. Any children with other disabilities, such as cerebral palsy, auditory neuropathy, syndromes, hypoplasia of the auditory nerve, or bilateral implant, were excluded.

All of the children with cochlear implants who were mainstreamed, and the deaf children without cochlear implants were placed in schools in which they were taught using sign language.

### National database

To compare sample parameters with the Portuguese population, we accessed a hospital admissions database, courtesy of the Central Administration of the Health System. The national database contains information such as anonymized patient identification, episode, process number, age, sex, admission date, discharge date, ward(s), hospital attended (tertiary vs. university), district, outcome (death, discharge, or transfer), and payment data (diagnosis related groups). It also contains ICD-9-CM codes for principal and secondary diagnoses (up to 19), procedures (up to 20), and external causes (up to 20). The patient population included all patients hospitalized in all acute care public hospitals in Portugal. The data were collected from 1992 to 2002 on children aged 8–18 years at the time of evaluation for cochlear implant placement. In this database, all implanted subjects were included because it was not possible to isolate prelingual deaf subjects. Therefore, we compared our sample with only those patients in the database who were hospitalized for implant placement at or before 5 years of age (i.e., the maximum age at the time of implant in the sample).

### Variable

School failure is measured with the percentage of repeated school years, i.e. the percentage of school years repeated calculated for each student. The sample population was characterized with respect to sex, age, socio-demographic status, hearing ability, etiology of deafness, age at diagnosis, cochlear implant, early intervention, sign language, preschool enrolment, deferred enrolment, and school reference.

### Study size

By contacting the Ministry of Education, a list of 10 schools that integrated children and adolescents with severe or profound deafness, with and without cochlear implants, was obtained. All of the schools were contacted, and a total of 7 schools agreed to participate in the study; however, only 5 met the inclusion criteria (i.e., the school was attend by both implanted and non-implanted deaf students as well as normal-hearing students). In the schools, 24 implanted and 24 non-implanted deaf students were identified as well as 24 normal-hearing students.

Informed consent was obtained from the school directors and the students’ parents.

### Data sources

The data on the number of school years failed were collected by the teachers based on the students’ school records. In addition, a questionnaire that included three questions regarding the level of a family’s participation in the student’s school life using a Likert scale was administered to the teachers.

While asking for parental consent, a semi-structured interview was administered to collect clinical histories and socio-demographic data. The Graffar Scale was used to determine socioeconomic status.

Access to the student records was conducted uniformly by teachers who followed a prescribed grid to minimize biases. In addition, a pilot study was conducted with 10 teachers to improve the questionnaire regarding family participation in the school life of the student.

### Ethics committee

This study was approved by the São João Health Centre Ethics Committee. All of the data collection was in accordance with the Helsinki Declaration of 1964, as revised in 2013.

### Statistical analysis

Kruskal–Wallis tests were used to determine if there was a different in the percentage of repeated school years among normal-hearing, implanted deaf, and non-implanted deaf students. The differences between the three groups were analyzed using Mann–Whitney tests and Bonferroni-adjusted *p* values. Chi square tests or Fisher exact tests were used to compare family participation in school life in the three groups. A statistical significance of 0.05 was used.

## Results

Seventeen of the 24 normal-hearing participants and 20 of the 24 selected participants with cochlear implants were included in the study. All others either chose not to participate or were excluded based on the exclusion criteria. Of 24 selected participants without cochlear implants all were included. Of the 61 participants, 35 (57 %) were female. Sixty-eight percent (n = 11) of the normal-hearing sample, 50 % (n = 10) of the cochlear-implanted deaf, and 58 % (n = 14) of the non-implanted deaf were female. There was no significant difference in the percentage of females among the three groups (*p* = 0.661). The mean (SD) age was 10 (3) years in the normal-hearing group, 11 (4) years in the implanted deaf group, and 13 (4) years in the non-implanted deaf group; these differences were not significant (*p* = 0.078). There were no significant differences between the 2 deaf groups regarding the etiology of the hearing disability. There were no significant differences observed between the three groups with respect to the socioeconomic status (*p* = 0.421). From the 61 participants, 7 % belong to the socioeconomic status I, 18 % belong to the socioeconomic status II, 57 % to the socioeconomic status III, 15 % to the socioeconomic status IV and 3 % belong to the socioeconomic status V. All of the participants were children of hearing parents and attended public school. Of the participants, 39 % were in the fourth grade, 25 % in the seventh grade, 18 % in the ninth grade, and 18 % in the twelfth grade. The group with cochlear implants was homogenous with respect to where the implant surgeries and post-implantation rehabilitations took place.

We compared the implanted study participants with deaf people in the Portuguese population who received implants between 1992 and 2002 and who were aged 8–18 years at the time of evaluation and 5 years old or less at the time of implant and found, no significant differences in sex (*p* = 0.662), age at implant (*p* = 0.345), or type of hospital where the implant was performed (*p* > 0.999). The only significant difference we found between these two groups was with respect to the district of residence (*p* < 0.001). Our participants were all from the same district (Porto), whereas those in the comparator population were from Porto (16 %), Aveiro (14 %), Lisbon (11 %), Braga (10 %), or other districts (48 %) (Table 1).

**Table 1:** Cochlear implants in children (5 years old or less) between 1992 and 2002: population and our sample characteristics

	Sample n = 20	Population n = 196	p
Female gender n (%)	10 (50)	88 (45)	0.662
Hospital			
Covões (Coimbra)	20 (100)	192 (98)	1.000
Sta Maria (Lisboa)	0	4 (2)	
Age of implant			0.416
1	0	4 (2)	
2	6 (30)	95 (48)	
3	10 (50)	66 (34)	
4	3 (15)	23 (12)	
5	1 (5)	8 (4)	
District			<*0.001*
Porto	20 (100)	32 (16)	
Aveiro	0	27 (14)	
Lisboa	0	22 (11)	
Braga	0	20 (10)	
Coimbra	0	16 (8)	
Outros	0	79 (40)	

*p* values <0.05 is presented in italic

Table 2 displays characteristics of the deaf participants based on the responses to the parent and teacher questionnaires. No significant differences were found between the deaf children who received implants and those who did not with respect to the areas studied. We found that 95 % of the implanted participants used both sign language and speech to communicate, whereas 5 % used speech only to communicate. All of the participants without implants used sign language. In either group, only 25 % of the students had at least one parent who used sign language. No significant differences were found between these two groups with respect to enrolment adjustments or adjustments in the evaluation process.

**Table 2:** Characteristics of the deaf children and adolescents with and without implants

	Deaf with implants *n* = 20	Deaf without implants *n* = 24	*p*
*Parents questionnaire:*			
Etiology of hearing loss *n* (%)			0.325
Genetic disorders	6 (30)	8 (33)	
Premature birth	0	1 (4)	
Meningitis	3 (15)	0	
Rubella	1 (5)	2 (8)	
Toxoplasmosis	1 (5)	0	
Unknown	9 (45)	13 (54)	
Diagnosis age (months) *median (min, max)*	21 (6, 36)	24 (0, 48)	0.866
Sign language use *n* (%)			
Participants	19 (95)	24 (100)	0.455
At least one parent	5 (25)	6 (25)	0.540
*Teachers questionnaire:*			
School reference^a^ *n* (%)	11 (55)	10 (42)	0.378
Was enrolled in preschool *n* (%)	18 (90)	22 (92)	1.000
Had an early intervention *n* (%)	8 (40)	8 (33)	0.647
Delay in enrolment *n* (%)	1 (5)	3 (12)	0.614
Special conditions of matriculation *n* (%)	18 (95)	19 (83)	0.363
Special assessment conditions *n* (%)	15 (79)	22 (96)	0.153

*p* < 0.05 is considered significant

^a^A network of reference schools for bilingual education of deaf students was established in 2008 to define the requirements needed to provide quality education for these students. The students in these schools benefit from teachers with specialized training in deafness and competence in sign language, deaf sign language teachers, sign language interpreters and speech therapists

**Fig. 1:**
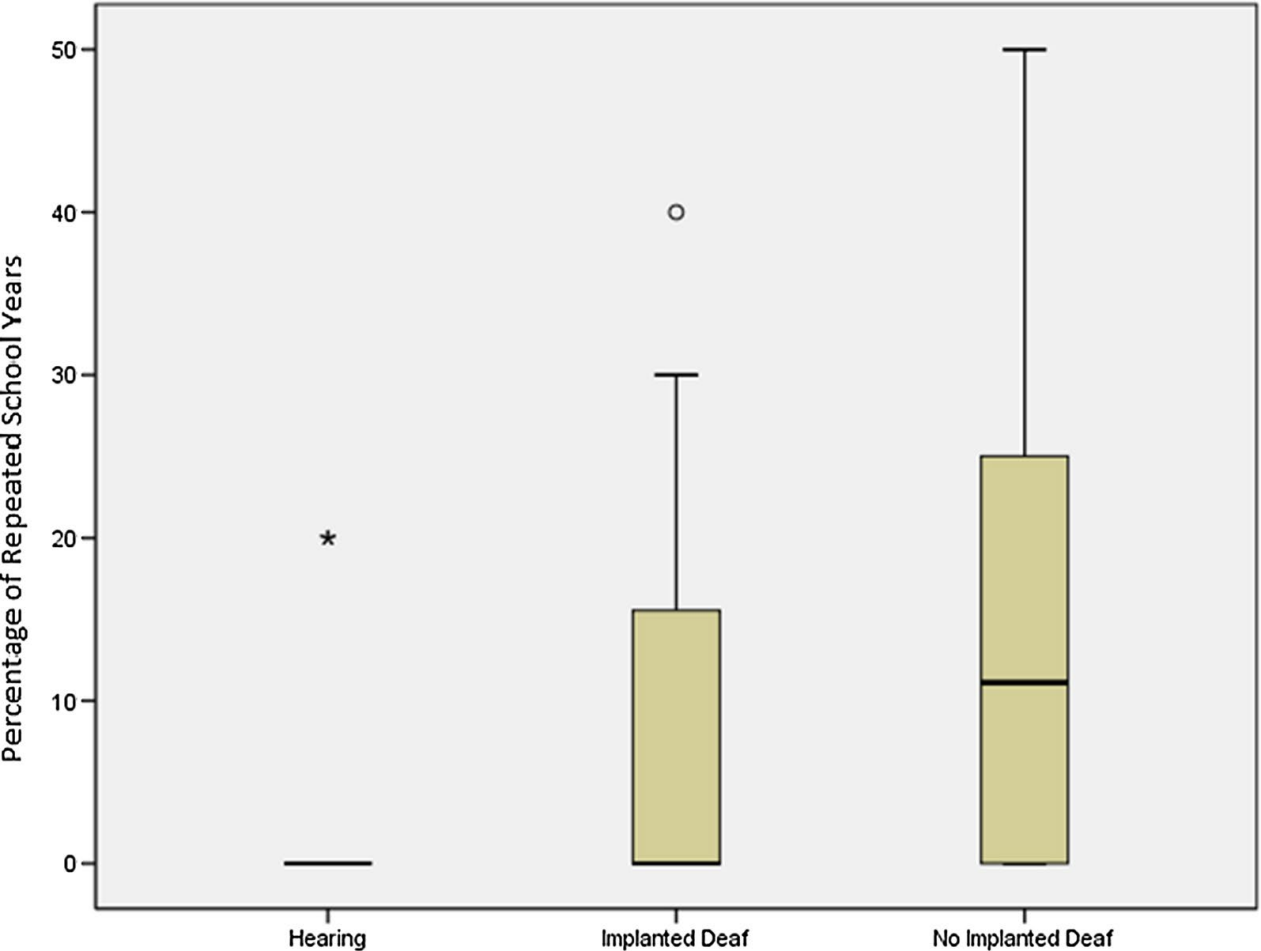
Median, interquartile range, minimum and maximum percentage of repeated school years per group (hearing, implanted deaf and no implanted deaf groups)

We found significant differences in the median percentage of school years repeated among the deaf implanted children, deaf children without implants and the normal-hearing children (*p* = 0.039). The median percentages were 0 % (range 0–20 %), 0 % (0–40 %), and 11 % (0–50 %) in the normal-hearing, deaf implanted, and deaf non-implanted groups, respectively (see Fig. 1). With respect to the median percentage of repeated school years, a significant difference was found between the normal-hearing and non-implanted deaf participants (*p* = 0.048), but no significant difference was found between the implanted deaf participants and those with normal-hearing (*p* = 0.675) or the non-implanted deaf participants (*p* = 0.423).

Table 3 shows that among the three groups, there was no significant difference in the frequency with which the guardian contacted the school or was concerned with the students’ progress as reported by the teachers. In contrast, teachers reported that guardians of non-implanted deaf students helped students with school work less often than guardians of non-hearing impaired or deaf implanted students.

**Table 3:** Family participation in the school life of the students as characterized by the teachers

Describe family participation in the school life of the student:	Total	Normal hearing	Deaf with Implants	Deaf without Implants	*p*
The guardian contacts the school *n* (%)					0.636
Always/almost always	39 (70)	11 (79)	12 (63)	16 (70)	
Sometimes/rarely/never	17 (30)	3 (21)	7 (37)	7 (30)	
The guardian is concerned with the student’s progress *n* (%)					0.630
Always/almost always	42 (75)	12 (86)	14 (74)	16 (70)	
Sometimes/rarely/never	14 (25)	2 (14)	5 (26)	7 (30)	
The guardian helps the student with school work *n* (%)					0.023*
Always/almost always	24 (43)	9 (64)	10 (53)	5 (22)	
Sometimes/rarely/never	32 (57)	5 (36)	9 (47)	18 (78)	

* p < 0.05

## Discussion

The finding that the median percentages of repeated school years of the normal-hearing, cochlear-implanted deaf students were similar and lower than that of deaf students without implants suggest that cochlear implants reduce the number of school failures, although the difference in between the deaf with implants and deaf without implants was not statistically significant.

The group of implanted deaf students in this study did not appear to be biased because of the studied characteristics, the only statistically significant difference between the selected participants and the deaf implanted Portuguese population was their district of residence; no significant difference in sex, age, or place of cochlear implantation was found. We believe that the district of residence is not a factor that biases the data from our sample. Although 11 cases were lost after the participants were age- and sex-matched, this did not bias our results because the groups remained comparable in sex, age, and socio-demographic status.

There were no significant differences between the non-implanted and implanted deaf study participants regarding enrolment adjustments or adjustments in the evaluation process. Both groups benefited from these special measures that aim to promote access, educational success, and equal opportunities.

Failure usually results from a combination of factors and can have lifelong consequences. Byrd (2005) stated that health conditions can impair academic performance, and one in five children who repeat a grade in school has some identifiable disability.

Deaf students without cochlear implants appear to fail more than deaf students with cochlear implants. Experience shows that worldwide, the non-implanted deaf are largely excluded from tertiary education (Ruben 2000). Lang (2002) stated that teachers need to be better prepared to teach deaf students, providing these students with quality elementary and secondary educational opportunities so that they have equal access to higher education.

Once science demonstrates that the learning capabilities of an individual are not determined at birth but rather are the result of life history, experience, and the wealth of stimuli offered by the environment, new perspectives and duties emerge. Thus, it is no longer only a question of equal access to school but one of equal knowledge (i.e., the necessary opportunities as well as the means should be given to all so that learning is possible for all).

Therefore, we believe that schools and society in general must tailor resources in a way that ensures that the right conditions exist to allow deaf children to develop personalities and skills. Unequal results are inevitable, but they are acceptable if these children have been afforded learning conditions of equivalent quality as their normal-hearing counter-parts.

Thus, the equality of opportunity reflects the need to ensure the normal performance, not necessarily the equal performance, of each individual. Every individual must have the necessary means to make a choice. Equality comprises, in this way, the concept of individual self-realization.

Allowing deaf people to become part of the community is only an initial step because being part of the community means being part of the structure and playing a social role. The real challenge is for deaf people to perform social functions that are valid and valued.

Moreover, cochlear implantation appears to favor the perception of a good quality of life in deaf children and adolescent compared with deaf peers without cochlear implant (Duarte et al. 2014). This finding reflects the satisfaction of the children and adolescents with their own competence and academic performance.

Here, the role of technicians and teachers may be relevant. Several authors have observed that schools are in the best position to take the initiative of approaching the family and community (Harry 1992; Shen et al. 1994). When parents are aware of what their children are learning, they are more likely to help or become involved in their child’s learning activities at home when requested by teachers to do so.

We were also able to investigate the effect of family participation on school life, although they were evaluated in an indirect way through the perspective of the teachers. It was found a significant difference between the three groups in respect to the support given by their guardians regarding homework. The teachers believed that the normal hearing children are the ones receiving more support, followed by the implanted children and lately the hearing impaired children with less support. This may be due to a lack of competence concerning sign language as well as a distrust of their capacities to help, we found that only 25 % of the deaf students with and without cochlear implants had at least one parent able to communicate in sign language (25 %). This effectively reduces or limits communication between these parents and their children, especially if this is the only method of communication.

Although there is no consensus in the literature on the subject, Lyness et al. (2013) found no convincing evidence that the use of sign language was detrimental to the success of the cochlear implant. On the contrary, the success of the cochlear implant seems to depend on audiovisual integration skills. Early placement of a cochlear implant is an amazing contributor to the acquisition of functional hearing for congenitally deaf children. However, language skills and cognitive development should not be overlooked when considering the effectiveness of a cochlear implant (Lyness et al. 2013). In this study, 95 % of the implanted deaf used both sign language and speech to communicate; 5 % used speech only.

Horacek et al. (1987) demonstrated that educational intervention reduced the incidence of grade failure most successfully (15 % reduction) when delivered both as preschool and school-age programs, and that achievement test scores in reading and mathematics showed a parallel beneficial effect from intervention. These data support the use of early intervention programs that target high-risk children as a mean of reducing their rate of school failure.

Undoubtedly, one of the current challenges of the educational community is the ability to facilitate successful learning in all students, regardless of their socioeconomic status, cultural or family situation, personality characteristics, abilities, or any type of deficit.

In this sense, every child or young person requires a proper analysis of their situation. Attention to individual differences requires the delivery of a personalized education to each student. Thus, it is the responsibility of the education system to fit into reality or rather to put into practice what is laid out in the various legal documents focused on the matter.

Thus, in a general sense, we can say that to achieve educational success, particularly of a deaf child, we should take into account from an early age the characteristics and particular needs of each student, realizing that the needs of an implanted deaf child will be different from those of a normal-hearing child or a deaf child without implants. On the other hand, the age of deafness onset, the time lag between diagnosis and initiating the rehabilitation process, and the home environment of a child are paramount in a child’s functional recovery. Thus, it is critical that deafness screening be promoted and conducted in an equitable manner on all newborns with the aim of identifying hearing loss so that rehabilitation can proceed in global and multidisciplinary terms as soon as possible.

## Conclusion

The results of the cochlear implanted children and adolescents are closer to the normal hearing children in respect to the percentage of school years repeated, compared to the hearing impaired children and adolescents with no implant. The teachers perceived that the parents of the normal hearing and of the implanted children and adolescents give more support regarding homework, in comparison to the parents of the hearing impaired children and adolescents with no implant.

Thus, the responsibility of parents, health professionals, teachers, and society as a whole should be proportional to their power, expressing a duty that is never merely individual but rather requires a broad political organization that follows and enforces it. Based on these results and the results of other more in-depth studies, in the future, it will be possible to identify with greater accuracy and precision the specific characteristics and factors influencing grade retention so that intervention programs can be tailored to the needs of deaf children and everyone can have an equal opportunity to fully achieve their potential within the same time period.
